# Impact of actin polymerization and filopodia formation on herpes simplex virus entry in epithelial, neuronal, and T lymphocyte cells

**DOI:** 10.3389/fcimb.2023.1301859

**Published:** 2023-11-24

**Authors:** Thanayod Sasivimolrattana, Parvapan Bhattarakosol

**Affiliations:** ^1^ Department of Microbiology, Faculty of Public Health, Mahidol University, Bangkok, Thailand; ^2^ Center of Excellence in Applied Medical Virology, Department of Microbiology, Faculty of Medicine, Chulalongkorn University, Bangkok, Thailand; ^3^ Division of Virology, Department of Microbiology, Faculty of Medicine, Chulalongkorn University, Bangkok, Thailand

**Keywords:** herpes simplex virus type 1 (HSV-1), viral entry, filopodia formation, actin polymerization, epithelial cell, neuron, T lymphocyte

## Abstract

Herpes simplex virus type 1 (HSV-1) has been known as a common viral pathogen that can infect several parts of the body, leading to various clinical manifestations. According to this diverse manifestation, HSV-1 infection in many cell types was demonstrated. Besides the HSV-1 cell tropism, e.g., fibroblast, epithelial, mucosal cells, and neurons, HSV-1 infections can occur in human T lymphocyte cells, especially in activated T cells. In addition, several studies found that actin polymerization and filopodia formation support HSV-1 infection in diverse cell types. Hence, the goal of this review is to explore the mechanism of HSV-1 infection in various types of cells involving filopodia formation and highlight potential future directions for HSV-1 entry-related research. Moreover, this review covers several strategies for possible anti-HSV drugs focused on the entry step, offering insights into potential therapeutic interventions.

## 1 Introduction

**Figure 1:**
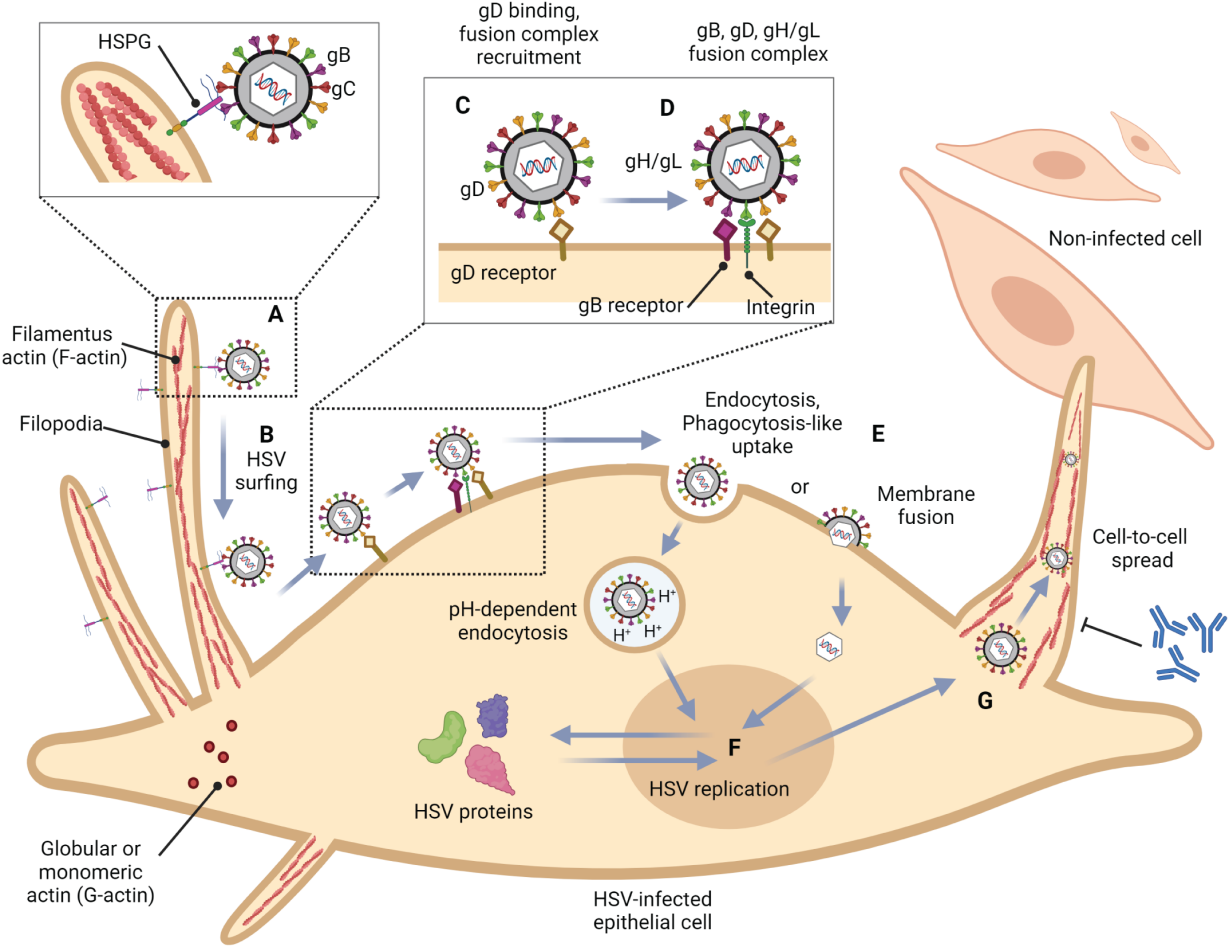
Role of filopodia formation in herpes simplex virus (HSV) infection in epithelial cells. **(A)** HSV gB and/or gC bind to HSPG which is present at the filopodia. This structure is constructed by the polymerization of G-actin to F-actin. **(B)** HSV surfs along filopodia to the cell surface. **(C)** The interaction of HSV gD with one of their receptors (Nectin-1, HVEM, and 3-OS HS) induces a conformational change. **(D)** The recruitment of a fusion complex including gB, gD, and gH/gL is induced. **(E)** HSV enters cells by fusion, or endocytosis. **(F)** HSV replication cycle **(G)** HSV infection can reorganize the actin cytoskeleton and induce filopodia formation to facilitate other HSV entry and spread the virion to another cell. This mechanism also promotes HSV to evade host immune responses.

Herpes simplex virus (HSV) is a widespread pathogen that has diverse clinical manifestations ranging from mild to severe diseases leading to death, for example, cold sores, herpetic whitlow, gingivostomatitis, and neonatal herpes (Berkovich and Ressel, 1966; Crane and Lerner, 1978; Pardo et al., 2004; Slifer and Jennings, 2015). HSV belongs to the family *Herpesviridae*, subfamily *Alphaherpesvirinae*. There are two types, i.e., HSV-1 and HSV-2 (Cliffe et al., 2014). HSV-1 causes oropharyngeal lesions and is transmitted by direct contact, while HSV-2 is a sexually transmitted disease that generally infects the genital mucosa (Brooks et al., 2013). HSV is a double-stranded DNA virus. Its genome is contained in an icosahedral capsid. The viral envelope comes from the host either nuclear membrane or cytoplasmic membrane, where various types of viral glycoproteins, for instance, glycoproteins (g) B, D, H, and L are located (Ahmad and Wilson, 2020). These glycoproteins are very essential for herpesvirus attachment and entry (Agelidis and Shukla, 2015). The well-known tissue tropisms of HSV are epithelial and mucosal cells. After HSV infection, the virus can cause latent infection by persisting in the neuronal ganglion (Brooks et al., 2013). Recurrent infection can occur from time to time when reactivation is induced by stimuli such as burns, colds, stress, and immunosuppressive drugs. During latency, the virus stays in the nerve cell without replication. Although HSV-1 normally infects epithelial cells, previous studies showed the possibility of HSV replication in a human T lymphocyte (Teute et al., 1983; Bhattarakosol et al., 2002). As a result, HSV entry mechanisms in T lymphocytes has been deeply explored in many previous works. In addition, it has been demonstrated that HSV induces filopodia formation, an actin-rich plasma membrane protrusion (**Figure 1**), to enhance infectivity in the entry step into various kinds of cells (Oh et al., 2010). In this review, the role of filopodia in HSV infection in diverse cell tropisms (epithelial, neuronal, and T cells) will be summarized.

## 2 HSV entry

To this date, the prototype of the HSV entry model is based on an epithelial cell platform (**Figure 1**). HSV requires various molecules including glycoprotein (g)B, gC, gD, gH and gL for entry into host cells. They interact with their specific molecules (receptors) successively. Firstly, HSV uses gB and/or gC, located on the envelope, to attach to the heparan sulfate proteoglycans (HSPG) which are expressed on the membrane of the target cell, called the attachment step. At this step, gC is also able to bind to HSPG; however, gC-deficient HSV can use only gB for attachment (Agelidis and Shukla, 2015). Alternatively, filopodia formation can increase the opportunity for HSV attachment (Oh et al., 2010). HSV surfing along the surface of these filopodia by using their gB attachment to HSPG causes actin remodeling (Clement et al., 2006). Oh et al. demonstrated that HSPG expression is increased on the filopodia, while the expression of the gD receptor is located only at the cell surface (Oh et al., 2010). Moreover, filopodia formation is induced by the interaction between HSV and the cell. The chances of other HSV particles binding are increased after the first HSV particle binds (Clement et al., 2006). After HSV arrives at the cell surface, it uses gD to bind to the gD receptor on the host cell surface (Agelidis and Shukla, 2015). There are three kinds of gD receptors expressed in different cell types, including Nectin-1 and 2, Herpes virus entry mediator (HVEM), and 3-O-sulfated heparan sulfate (3-OS-HS) (**Table 1**). After gD binds to one of its receptors, a conformational change in this glycoprotein’s structure drives the recruitment of a fusion complex including gB, gH, and gL (Cairns and Connolly, 2021; Connolly et al., 2021; Atanasiu et al., 2023). gB, a member of class III fusogen (Heldwein et al., 2006), binds to one of its receptors to initiate membrane fusion. Recently, it was revealed that gB interacts with multiple sites on gH to promote membrane fusion (Fan et al., 2023). There are three types of gB receptors (**Table 2**), including paired immunoglobulin-like receptor (PILRα), an inhibitory receptor found on macrophages, dendritic cells, and monocytes (Satoh and Arase, 2008), myelin-associated glycoprotein (MAG) which expresses on glial cells (Suenaga et al., 2010), and non-muscle myosin heavy chain-IIA (NMHC-IIA) on human tissue (Agelidis and Shukla, 2015). As mentioned above, gH and gL are also essential for HSV entry. These glycoproteins bind to αvβ6- and αvβ8-integrins to trigger the entry mechanisms (Gianni et al., 2013). HSV envelope fusion with the plasma membrane via either a pH-independent or pH-dependent process requires gB, gD, gH, and gL (Cai et al., 1988; Johnson and Ligas, 1988; Forrester et al., 1992; Roop et al., 1993; Nicola and Straus, 2004; Dollery et al., 2010). Pataki Z. and co-workers suggested that this glycoprotein complex is formed prior to and during membrane fusion (Pataki et al., 2022). This fusion complex leads HSV to merge its envelope with the lipid bilayer of the host membrane and release viral nucleocapsid and tegument proteins into the host cytoplasm (Agelidis and Shukla, 2015). Then, cell-to-cell fusion after HSV-1 and HSV-2 entry is induced by gD, gB, and gH/gL (Turner et al., 1998; Muggeridge, 2000; Pertel et al., 2001). Alternatively, HSV can enter the cell by endocytosis and phagocytosis-like uptake (Nicola and Straus, 2004; Clement et al., 2006). HSV binds to the gD receptor, which is localized in the endosome for fusion with the endosomal membrane (Clement et al., 2006). HSV-1 and HSV-2 can enter HeLa cells (human cervical cancer cells) and CHO cells (Chinese hamster ovary cells) expressing gD receptors with low pH-dependent endocytosis (Nicola et al., 2003). In some cells, such as human epidermal keratinocytes, gC regulates low-pH fusion machinery for HSV entry (Komala Sari et al., 2020). The phagocytosis-like uptake is induced by Rho GTPase activation and cytoskeleton rearrangement. The pathway that HSV uses for entry depends on cell types (Stefan Pohlmann, 2013). HSV prefers fusion with the plasma membrane to enter Vero cells (monkey kidney epithelial cells), human neurons, human foreskin fibroblasts, and Hep-2 cells (Lycke et al., 1988; Nicola et al., 2005; Nicolai et al., 2016). On the other hand, HSV enters retinal pigment epithelial cells, human conjunctival epithelial cells, human epidermal keratinocytes, and HeLa cells by endocytosis (Nicola and Straus, 2004; Akhtar and Shukla, 2009). In conclusion, gB, gD, and gH/gL are essential for both HSV entry pathway (Milne et al., 2005; Nicolai et al., 2016). After HSV enters the cell, the viral nucleocapsid and tegument proteins travel along the cytoplasm to the nucleus with the help of actin and myosin (Favoreel et al., 2007). Once the HSV’s genome reaches the nucleus, the HSV replication cycle is initiated.

**Table 1:** gD receptors of HSV.

gD receptors		Info	Expressing on	Notes
Nectin	Nectin-1	- Members of the immunoglobulin superfamily - Play a role in cell adhesion	- Epithelial cells, Endothelial cells, Neuronal cells, Keratinocytes (Geraghty et al., 1998; Warner et al., 1998; Karasneh and Shukla, 2011)	- Cells expressing Nectin-2 need more HSV particles than Nectin-1 for infection (Lopez et al., 2000). - Nectin-1 or -2 can mediate HSV-2 entry, but wide-type HSV-1 can interact with nectin-1 only (Krummenacher et al., 2004). - The amino acid sequence of Nectin-1 is 30% different from Nectin-2.
	Nectin-2		- Epithelial, Endothelial, Neuronal cells (Karasneh and Shukla, 2011)	
HVEM (Herpes virus entry mediator)		- Also known as herpes virus entry protein A (HVEA) - Member of the tumor necrosis factor (TNF) receptor superfamily (Montgomery et al., 1996; Kovacs et al., 2009)	- Ocular epithelial cells - Many immune cells, e.g., natural killer (NK) cells, B, and T lymphocytes, dendritic cells (DC), macrophages, polymorphonuclear, and myeloid cells (Kwon et al., 1997; Edwards and Longnecker, 2017). - Fibroblasts, Epithelial cells, Endothelial cells (Hung et al., 2002)	- HVEM also plays a role in HSV latency and reactivation (Allen et al., 2014a). - Natural ligands: - BTLA (B- and T-cell attenuator) (Compaan et al., 2005). - LIGHT (receptor expressed on T lymphocytes that has an effect on regulating lymphocyte activation) (Mauri et al., 1998; Croft, 2005) - Ig-like ligand (CD160) (Edwards and Longnecker, 2017)
3-OS HS (3-O-sulfated heparan sulfate)		- The polysaccharide, which contains various modifications of heparan sulfate (O'Donnell and Shukla, 2009). - Heparan sulfate (HS) is modified by the 3-OST (3-O-Sulfotransferase) family to turn to 3-OS HS, modified at the C3 position of the glucosamine precursor, as a gD receptor (Xia et al., 2002; Tiwari et al., 2004; Xu et al., 2005; O'Donnell et al., 2006; Tiwari et al., 2015).	- Primary culture of corneal fibroblast cell (Tiwari et al., 2006). - Zebrafish model (Yakoub et al., 2014) - Some kinds of human tissue and cell line (less than other gD receptors), i.e., liver, placenta, heart, kidney, pancreas and human epithelial cell (Campadelli-Fiume et al., 2000).	- This gD receptor is an important receptor for human herpesviruses attachment, except for EBV (Shukla and Spear, 2001). - 3-OS HS not only induces HSV-1 entry but also cell-cell fusion (Tiwari et al., 2004; Tiwari et al., 2007). - gD recertor for HSV-1 (HSV-2 gD cannot bind with 3-OS-HS) (Shukla et al., 1999).

**Table 2:** gB receptors of HSV.

gB receptors	Info	Expressing on	Notes
Paired Immunoglobulin-like type 2 receptor-α (PILRα)	- Members of the PILR family are significant surface molecules that interact with ligands to modulate the host immune response (Lu et al., 2014)	- Mainly expressed on various types of immune cells, e.g., myeloid cells, monocytes, macrophages, and dendritic cells (Satoh et al., 2008; Arii and Kawaguchi, 2018).	- HSV resistant cells that express the PILR are susceptible to almost all alpha-herpesviruses, with the exception of HSV-2 (Arii et al., 2009).
Myelin-Associated Glycoprotein (MAG)	- The paired receptor family MAG shares 5-12% homology with PILR (Suenaga et al., 2010)	- Glial cells (Arii and Kawaguchi, 2018)	- gB receptor of HSV-1 and varicella-zoster virus (VZV) (Suenaga et al., 2010). - Since MAG is not expressed in epithelial cells, it is not the primary receptor for the HSV (Madavaraju et al., 2020).
Non-Muscle Myosin Heavy Chain IIA (NMHC-IIA)	- A subunit of non-muscle myosin IIA (NM-IIA) (Madavaraju et al., 2020).	- Epithelial cells, endothelial cells, and neurons	- NM II binds to actin in the host cell and controls typical cellular processes like cell division, adhesion, movement, migration, and contraction (Karasneh and Shukla, 2011; Agelidis and Shukla, 2015)

### 2.1 Actin polymerization and dynamics

As mentioned, filopodia formation plays a critical role in HSV infection in epithelial cells, their tissue tropism (**Figure 1**). Filopodia are actin-rich structures that contain strongly bound horizontal actin bundles that are polymerized in this structure, called filamentous actin (F-actin) polymerization. In mammals, there are 6 actin genes (*Acta1, Acta2, Actc1, Actb, Actg1*, and *Actg2*), each gene encodes one protein isoform, including α_skeletal_-actin, α_smooth_-actin, α_cardiac_-actin, ß_cyto_-actin, γ_cyto_-actin, and γ_smooth_-actin, respectively. The amino acid sequences of these isoforms have more than 93% similarity (Perrin and Ervasti, 2010). There are two basic types of actin that localize in various cells: globular or monomeric actin (G-actin) and linear polymer or filamentous actin (F-actin) (Roberts and Baines, 2011). Both are essential for cell functions such as contraction of cells during cell division and filopodia formation (Mattila and Lappalainen, 2008; Simiczyjew et al., 2017). The dimensions of actin monomer are about 5.5 x 5.5 x 3 nm (Sonja Kuhn, 2017). F-actin, the physiologically active form of actin, is obtained by the polymerization of G-actin monomers (Lodish et al., 2000; Perrin and Ervasti, 2010). The F-actin polymerization process is divided into 3 steps, including nucleation, elongation, and steady state (Lodish et al., 2000). First, nucleation phase, G-actin is bound by one molecule of Adenosine triphosphate (ATP) to maintain its native configuration. After that, a stable complex of actin is obtained by gathering three molecules of ATP-G-actin monomers. Second, elongation phase, ATP-G-actin monomers are added at both ends of the filament, after G-actin interaction in a growing filament, the bound ATP is hydrolyzed rapidly and turned to adenosine diphosphate (ADP) and inorganic phosphate (Pi). Because of the structural polarity of F-actin, the polymerization rates at both ends of the filament are different. The fast-growing end is called the barbed end (+), whereas the slow-growing end is called the pointed end (-). Third, steady state, after the polymerization process of G-actin to F-actin, the ATP-G-actin turns into a stable ADP-F-actin by ATP hydrolyzing. In this step, G-actin molecules exchange with the filament ends without increasing the total F-actin amount. The F-actin has a diameter of about 8 to 10 nm and contains about 1,000 G-actin monomers along its 1 µm length (Sonja Kuhn, 2017). The properties of F-actin depend on the isoforms that mix in the filament (Perrin and Ervasti, 2010). The polymerization and depolymerization rates of ß_cyto_-actin are faster than those of γ_cyto_-actin. However, both isoforms can copolymerize together, and the polymerization rates depend on ß_cyto_-actin and γ_cyto_-actin ratios (Bergeron et al., 2010).

There are three basic types of actin networks that are caused by actin polymerization: stress fibers, filopodia, and lamellipodia. Stress fibers are networks which constructed by the arrangement of F-actin bundles with alternating polarity. These filaments are combined via interactions between the dimeric bundling protein α-actinin and the motor protein myosin II. Stress fibers play a role in maintaining cell attachment to the platform and changing cell morphology. Filopodia formation consists of compact and strongly bound parallel F-actin bundles that polymerize along the cell membrane, forming a spike-like structure (Roberts and Baines, 2011). This structure plays an important role in the processes of wound healing and extracellular matrix adhesion (Welch and Mullins, 2002). Moreover, the function of this formation is as a sensor of the extracellular environment, which contains various receptors for signaling, for example, cell adhesion molecules including integrins and cadherins. Lamellipodia consist of actin networks that extremely branch near the cell membrane. The emanation of these formations is initiated by the activation of Rho GTPase (Roberts and Baines, 2011). These proteins are regulated by Guanosine triphosphate/Guanosine diphosphate (GTP/GDP) binding; GTP-bound is an active form, whereas GDP-bound is an inactive form (Hall, 1998; Etienne-Manneville and Hall, 2002). There are three extensive Rho family members involved in actin rearrangement, including Ras homolog gene family, member A (RhoA), Ras-related C3 botulinum toxin substrate 1 (Rac1), and Cell division control protein 42 (Cdc42). RhoA plays a role in the gathering of F-actin to produce stress fibers. Rac1 is associated with lamellipodia and ruffling. Cdc42 is essential for filopodia formation (Roberts and Baines, 2011). Moreover, Rho GTPase is involved in phagocytosis uptake (Hall, 1998). Phagocytosis uptake requires receptors, e.g., the Fc receptor (FcR), to mediate actin rearrangement and to transport actin to the site of stimulating particle (Chimini and Chavrier, 2000).

### 2.2 HSV entry into epithelial cells

Although it has been demonstrated that the cells get several benefits from filopodia formation, some pathogens, especially viruses, can hijack the presence of this structure to support their life cycle (**Figure 1**). F-actin is remodeled along the viral life cycle (entry, assembly, and egress) by the activation of RhoA, Cdc42, and Rac1 (Taylor et al., 2011). Filopodia formation is regulated by Cdc42 to induce ARP2/3 complex actin filaments (Mattila and Lappalainen, 2008; Chang et al., 2016). Cdc42 is also activated by the interaction of HSV-1 with epithelial cells, leading to filopodia formation. Oh et al. investigated that filopodia are induced in Vero cells after exposure to HSV-1 for 15 minutes. Filopodia formation and HSV-1 entry are decreased by Cdc42 down-regulation (Oh et al., 2010). Another Rho GTPase protein, Rac-1, is activated together with Cdc42 during HSV-1 infection in fibroblasts and epithelial cells (Quetglas et al., 2012). After filopodia are induced via HSV-1 attachment, other HSV-1 particles bind to their receptor, HSPG, which expresses on filopodia and surfs along these structures to go to the cell surface. At the early stage of HSV-1 infection, F-actin assembly rates are increased (Chang et al., 2016). Actin cytoskeleton rearrangements promote not only HSV-1 infection by filopodia formation but also HSV-1 transport to the cell body and nucleus (Xiang et al., 2012). Besides HSV, human immunodeficiency virus (HIV) and human papillomavirus (HPV) can also surf along filopodia formation (Chang et al., 2016). Sometimes filopodia are retracted after viruses, such as HPV, bind to this structure to promote viral entry (Smith et al., 2008). It has been reported that many viruses, i.e., Epstein-Barr virus (EBV), human herpesvirus-8 (HHV-8), influenza virus, respiratory syncytial virus, HIV, and dengue virus type 2 (DENV-2), activate Cdc42 to induce actin cytoskeleton rearrangement to facilitate viral entry (Puls et al., 1999; Sharma-Walia et al., 2004; Zamudio-Meza et al., 2009; Nikolic et al., 2011; Wang et al., 2012; Xiang et al., 2012; Krzyzaniak et al., 2013). Filopodia can be used as a marker for viral infection. The amount of these actin-rich structures increases during HSV-1, CMV, or HHV-8 infection (Chang et al., 2016). Moreover, viruses can travel along filopodia to be transported to another cell. HSV gE, gI, and/or gK are essential for cell-to-cell spread (Farnsworth and Johnson, 2006; David et al., 2008). There are several advantages to viral spreading by cell-to-cell contact. The duration of viral spread by cell-to-cell spreading is faster than cell-free spreading. Another benefit is immune evasion due to the absence of viral antigens present in the environment (**Figure 1**). This suggests that neutralizing antibodies cannot work because there are no free viruses outside the cell (Mothes et al., 2010).

### 2.3 HSV entry into neurons

The stratified squamous epithelium (epidermis) of the oral and anogenital mucosa is a primary site of HSV infection. This infection is usually asymptomatic. After primary infection at the skin or mucosa, HSV infects sensory nerves and undergoes retrograde axonal transport to the neuronal cell body. Then, a life-long latent infection at the trigeminal and dorsal root ganglia (DRG) is established (Steiner et al., 2007; Kramer and Enquist, 2013). At the reactivation stage, HSV-1 undergoes anterograde axonal transport to the peripheral epithelial cells, leading to mild oral or labial lesions (Gilden et al., 2007). In addition to these symptoms, the virus can be asymptomatically shed (Tummon et al., 1981).

The main receptor for HSV-1 entry into neurons is nectin-1 (also known as HVEC and CD111) (Richart et al., 2003; Simpson et al., 2005). However, HVEM (also known as HVEA) also plays a role in HSV latency and reactivation. In the latency mice ocular model, Latency-associated transcript (LAT), a HSV-1 gene transcript abundantly expressed throughout latency, enhances HVEM expression (but not other gD receptors) (Allen et al., 2014a). Moreover, the amount of HSV-1 DNA in the trigeminal ganglion (TG) of wide-type (WT) mice infected with LAT (+) HSV-1 is higher than that of those infected with LAT (-) HSV-1. Additionally, the number of HSV-1 latent genomes from HVEM knockout mice that were infected with LAT (+) HSV-1 is lower than that of WT mice infected with LAT (+) HSV-1 (Allen et al., 2014b). Recently, it has been discovered that LAT produces two small non-coding RNAs (sncRNAs) that can control the expression of HVEM by stimulating its promoter (Tormanen et al., 2022). This indicates that LAT induces the overexpression of HVEM, leading to an increase in HSV-1 latency. In addition, HSV-1 reactivation might also increase.

**Figure 2:**
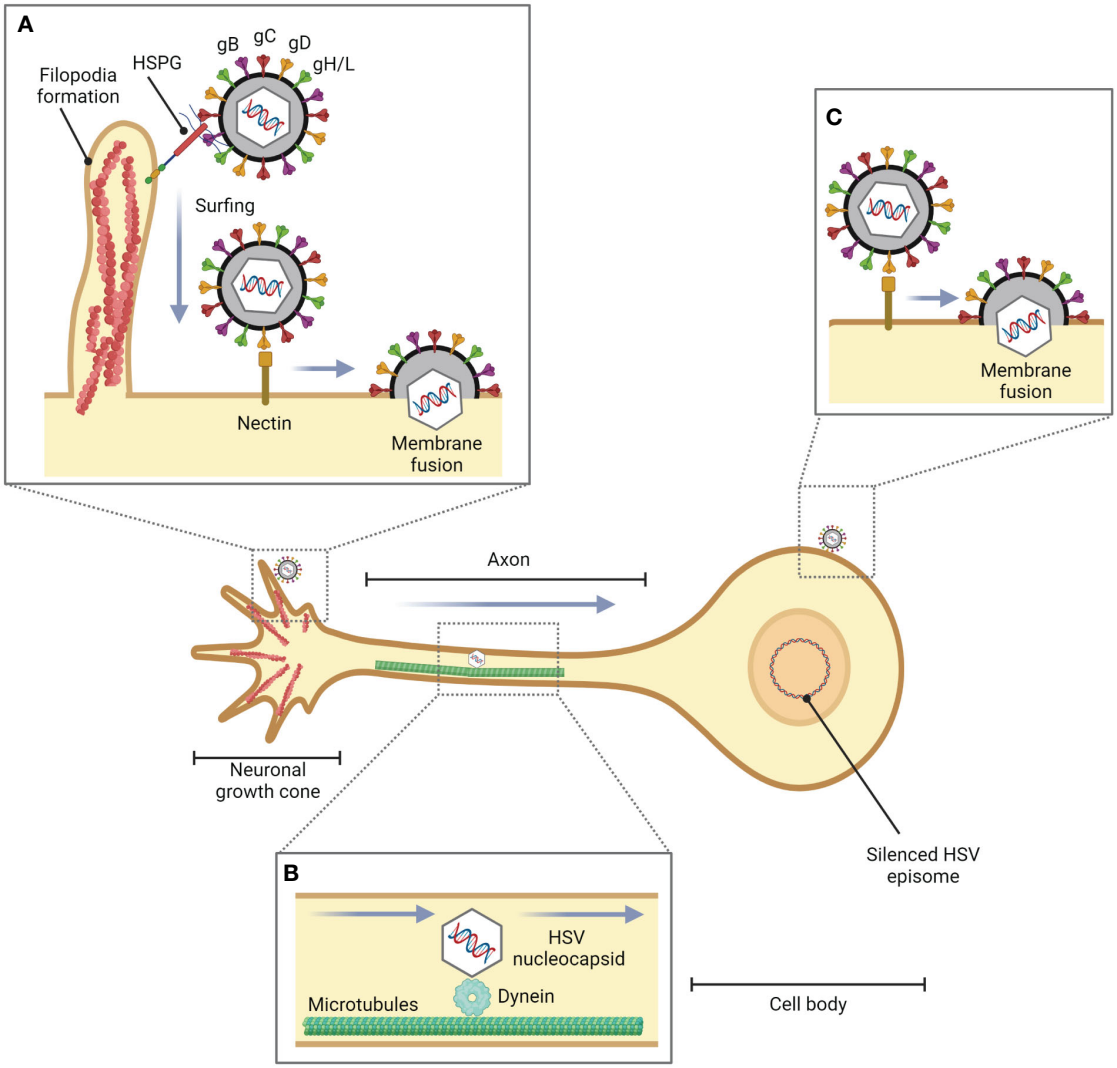
Role of filopodia formation in HSV entry into neuronal cells. During HSV infection in epithelial cells or fibroblasts, HSV is translocated from the site of infection to neurons. **(A)** HSV facilitates filopodia formation at the neuronal growth cone, a large actin-supported extension located at the tip of a developing or regenerating axon, and enters the neuronal cells by pH-independent membrane fusion via nectin as a gD receptor. **(B)** The HSV nucleocapsid is transported to the cell body by dynein, a cytoskeletal motor protein that plays a critical role in intracellular transport within eukaryotic cells, and microtubules. **(C)** HSV can also enter the cell body of neurons (*in vitro*).

The axon terminus near peripheral epithelial cells is the initial site of HSV-1 entry into the neuron (Antinone and Smith, 2010). However, it has been found that in the *in vitro* experiment, HSV-1 can enter the cell body by membrane fusion (Aggarwal et al., 2012). Additionally, filopodia not only facilitate HSV-1 entry in non-neuronal cells but also in neuronal cells (**Figure 2**). It has been investigated that Cdc42, RhoA, and Rac1 involved in HSV-1 entry in neuronal cells (De Regge et al., 2006). HSV-1 enters neuronal cells via the processes mediated by gB, gD, and gH/gL, as well as in epithelial cells. Unlike the HSV-1 entry mechanisms in human epidermal keratinocytes, pH-dependent endocytosis or the nectin-1 receptor-dependent membrane fusion pathway under low temperature (Sayers and Elliott, 2016), HSV-1 enters neuron by pH-independent fusion of its envelope with the neuronal plasma membrane (Nicola et al., 2005; Salameh et al., 2012; Sharthiya et al., 2017). During fusion, HSV-1 releases its icosahedral capsid and tegument proteins into the cell. Suddenly, the F-actin network plays a role as a physical barrier to hamper the entry of the virus. However, HSV-1 has evolved mechanisms to hijack this actin cytoskeleton to facilitate their entry and replication in the host cell by using inner tegument proteins, i.e., pUL36, pUL37, and pUS3, which are associated with viral capsid travel along the actin cytoskeleton and microtubules towards the nucleus in the cell body (Miranda-Saksena et al., 2018).

### 2.4 HSV entry into T lymphocytes

T lymphocytes are involved in the regulation of the immune response and in cell-mediated immunity. These cells recognize antigens and are activated to perform effective functions that respond to antigens, such as microorganisms. Naïve T lymphocytes arrive at secondary lymphoid organs, where they interact with antigen presenting cells (APC) and thus become activated. This activation process requires three signals. First, the interaction of MHC molecules, in the form of peptide-MHC complexes, on APC, e.g., dendritic cells, and T cell receptor (TCR); second, APCs are induced to express costimulatory molecules, for example, B7 proteins. These molecules interact with costimulatory receptors, CD28 molecules, which express on the T cell to provide second signals to naïve T cells. Third, the cytokine, interleukin-2 (IL-2), drives autocrine signals to activate T lymphocytes by interacting with its receptors on the T cell. Activated T cells are differentiated into effector or memory cells (Mak and Saunders, 2006). T lymphocyte activation can be occurred *in vitro* via nonspecific interactions with lectins, for instance, phytohemagglutinin (PHA), pokeweed mitogen (PWM), and concanavalin A (Con A), and can also be mimicked via the interaction of TCR and co-stimulatory receptors with specific antibodies, e.g., anti-CD3 and anti-CD28 antibodies (Trickett and Kwan, 2003; Shipkova and Wieland, 2012).

**Figure 3:**
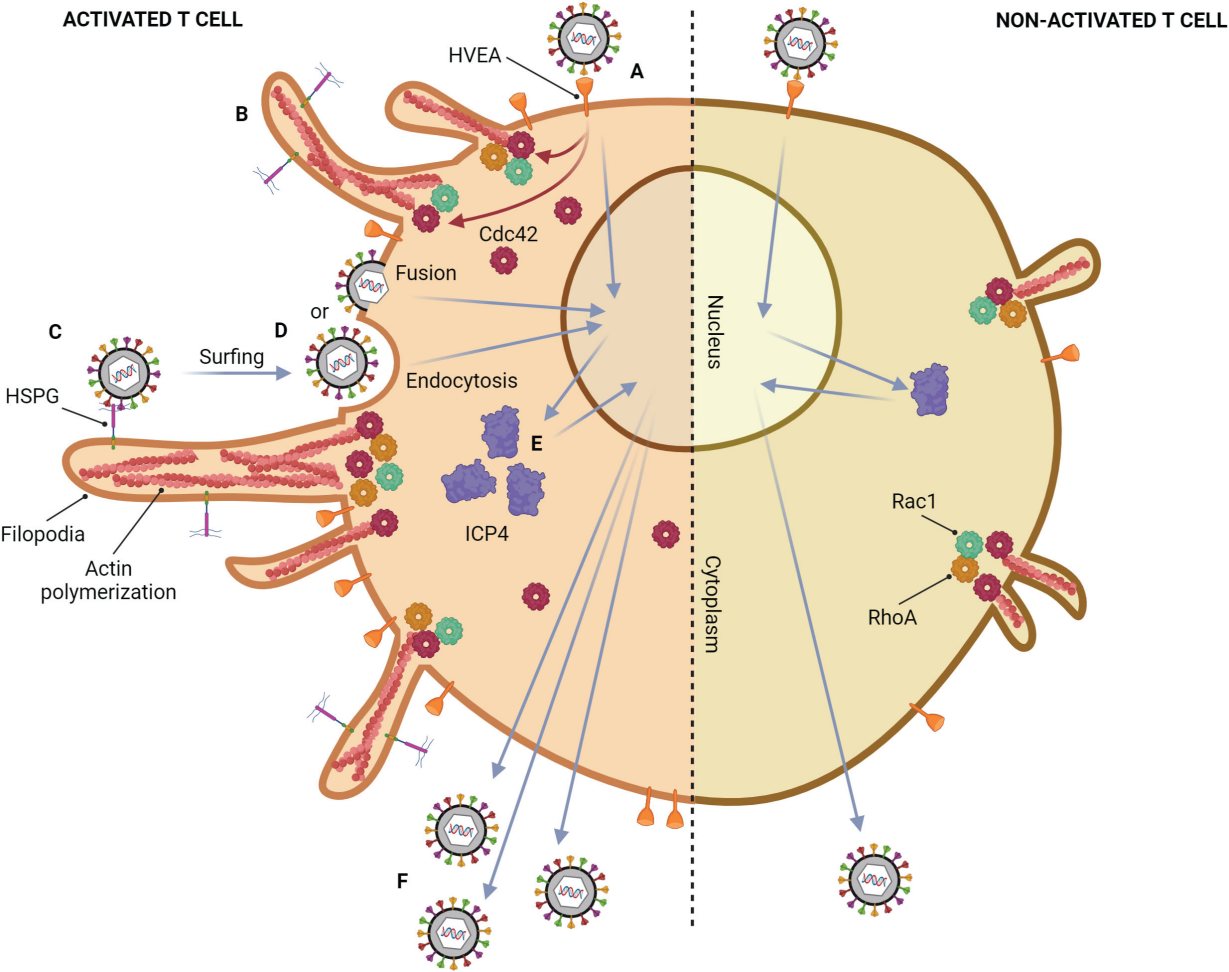
Mechanism of HSV-1 infection in a T lymphocyte. Activated T cell; **(A)** HSV-1 attaches to HVEM on the cell membrane, which is highly expressed after activation. **(B)** After binding, HSV induces filopodia formation via up-regulation of actin polymerization. This phenomenon might be regulated by the upregulation of Cdc42. **(C)** Other HSV particles attach to heparan sulfate proteoglycans (HSPG) which might be expressed on filopodia, and surf toward the cell body. **(D)** The viral particles enter the cell by endocytosis and membrane fusion. **(E)** ICP4 mRNA expression in activated T lymphocytes is 1 hour faster than that in non-activated T lymphocytes, leading to up-regulation of ICP4 protein. **(F)** The more HSV-1 entry and replication, the more HSV-1 production. Non-activated T cell; only small filopodia are found during HSV-1 attachment.

Although the common tissue tropism of HSV is epithelial, and mucosal cells, HSV can also infect T lymphocytes (**Figure 3**) (Rinaldo et al., 1978; Teute et al., 1983; Braun et al., 1984; Bhattarakosol et al., 2002). Previous studies show that HSV can replicate in Jurkat cells, an immortalized line of human T lymphocyte cells, but its production from T lymphocytes is lower than those from epithelial cells, Vero cells, and HEp-2 cells (Bhattarakosol et al., 2002). However, the number of HSV-1-infected T lymphocytes increases when the cells are activated by phytohemagglutinin (PHA), a mitogen-inducing activation and proliferation of lymphocytes (Chimma et al., 2004). HSV replication in non-activated T lymphocytes is delayed by at least 2 hours, and the production of HSV-1 from T lymphocytes is higher than HSV-2 (Bhattarakosol et al., 2002). HVEA, a HSV gD receptor that presents in T lymphocytes, is overexpressed after PHA activation, but HVEA mRNA expression cannot be detected in non-activated T lymphocytes (Chimma et al., 2004). Another study found HSV-1 production from anti-CD3/28-activated T lymphocytes is higher than that from non-activated cells (Bhattarakosol and Donchai, 2015; Sasivimolrattana et al., 2021). These results suggest that HSV-1 can be spread in the blood through T lymphocytes and cause viremia. In addition, HSV-1 and HSV-2 can evade immune responses by inducing apoptosis in HSV-infected T lymphocytes via a caspase-dependent pathway (Pongpanich et al., 2004). Moreover, after adsorption of HSV-1, ICP4 mRNA expression (an immediate early (α-) protein of HSV) in activated T lymphocytes is 1 hour faster than that in non-activated T lymphocytes, and ICP4 protein expression is up-regulated in activated T lymphocytes (Bhattarakosol and Donchai, 2015). Activating T lymphocytes with PHA or anti-CD3/28 antibodies may mimic *in vivo* situations, such as HSV coinfection in a patient who already has a latent stage caused by other organisms. In HIV infection, T cell activation is induced (Mahalingam et al., 1993; Biancotto et al., 2008). A previous study demonstrated that the percentage of CD3^+^ and CD38^+^ T lymphocytes from HIV-infected individuals is significantly different when compared with healthy donors. Since their T cells are already activated, HSV-1 production in T cells obtained from HIV-infected individuals is significantly higher than that from healthy donors. Moreover, both CD4^+^ and CD8^+^ T lymphocytes are shown to be susceptible to HSV-1 replication (Yamsuwan et al., 2012). In addition, one of the mechanisms that might support HSV-1 infection in activated T lymphocytes is filopodia formation (Bhattarakosol and Donchai, 2015). It has been demonstrated that filopodia formation by actin polymerization plays an important role in HSV-1 infection in various cell types, e.g., Vero, HeLa, RPE (human retinal pigment epithelium), ZF-3-OST-3 (zebrafish cells), Differentiated P19 (neural cells from mice), HCjE (human conjunctival epithelial cells), and CHO-K1 cells (Akhtar et al., 2008; Dixit et al., 2008; Tiwari et al., 2008; Oh et al., 2010; Tiwari and Shukla, 2010; Choudhary et al., 2013; Burnham et al., 2016). This actin polymerization plays a role in the formation of filopodia for HSV surfing, HSV entry, and HSV production (Dixit et al., 2008; Tiwari et al., 2008; Choudhary et al., 2013; Burnham et al., 2016). Interestingly, it was found that HSV-1 can also induce filopodia formation in activated T lymphocytes through actin polymerization as well as in epithelial cells (Sasivimolrattana et al., 2021). The study suggested that filopodia formation in activated T lymphocytes is possibly stimulated through the Cdc42 signaling pathway since upregulation of Cdc42 protein expression was found; however, there was no data about the active form of Cdc42 in activated T lymphocytes. A functional assay of Cdc42 activation leading to filopodia formation should be conducted in future studies. The presence of filopodia in activated T lymphocytes enhanced the viral entry into the cells, resulting in an increase in HSV-1 production in activated T lymphocytes. In addition, from the electron micrograph, it can be noticed that HSV-1 entry into activated lymphocytes might occur via endocytosis and fusion. Moreover, the virion production in T lymphocytes was mainly extracellular (Sasivimolrattana et al., 2021). The mechanism of HSV-1 infection in T lymphocytes is demonstrated in **Figure 3**. In addition to T cells, HSV-1 infection in human B cells has been investigated in a previous study. The research found that several cellular structures of B cells, such as cytoplasmic stress granules and multivesicular structures, were changed during HSV-1 infection (Chen et al., 2022). Nevertheless, there was no data about HSV-1 inducing actin polymerization and filopodia formation in B cells.

### 2.5 HSV entry into other cell types

Since HSV can infect a range of cell types, the relationship between filopodia formation and HSV entry in fibroblasts was observed in some studies. HSV-1 uses 3-OS HS as the receptor for entry into primary human corneal fibroblasts (CF) (Tiwari et al., 2006). The interaction between HSV and this receptor induces the actin cytoskeleton rearrangement, leading to the formation of filopodia. Pre-treatment with the actin-depolymerizing agent showed a negative effect on HSV entry in CF (Clement et al., 2006). In addition, it has been demonstrated that Rac-1 and Cdc42, the key molecules responsible for the activation of filopodia formation, are activated during HSV-1 infection in fibroblasts (Quetglas et al., 2012). Taken together, based on these evidences, HSV also induces filopodia in fibroblasts to support viral attachment and entry, similar to the epithelial cells.

Upon systemic infection, HSV-1 replicates in the endothelial cells (Doshi et al., 2023). In this cell type, HSV uses Nectin and HVEM as the gD receptors (Hung et al., 2002; Karasneh and Shukla, 2011). Although it has been reported that the endothelial cells are able to form the filopodia, there is no data about HSV inducing and surfing this filopodia in this type of cell. Future studies need to be conducted to observe this phenomenon.

## 3 HSV entry as an anti-viral target

**Figure 4:**
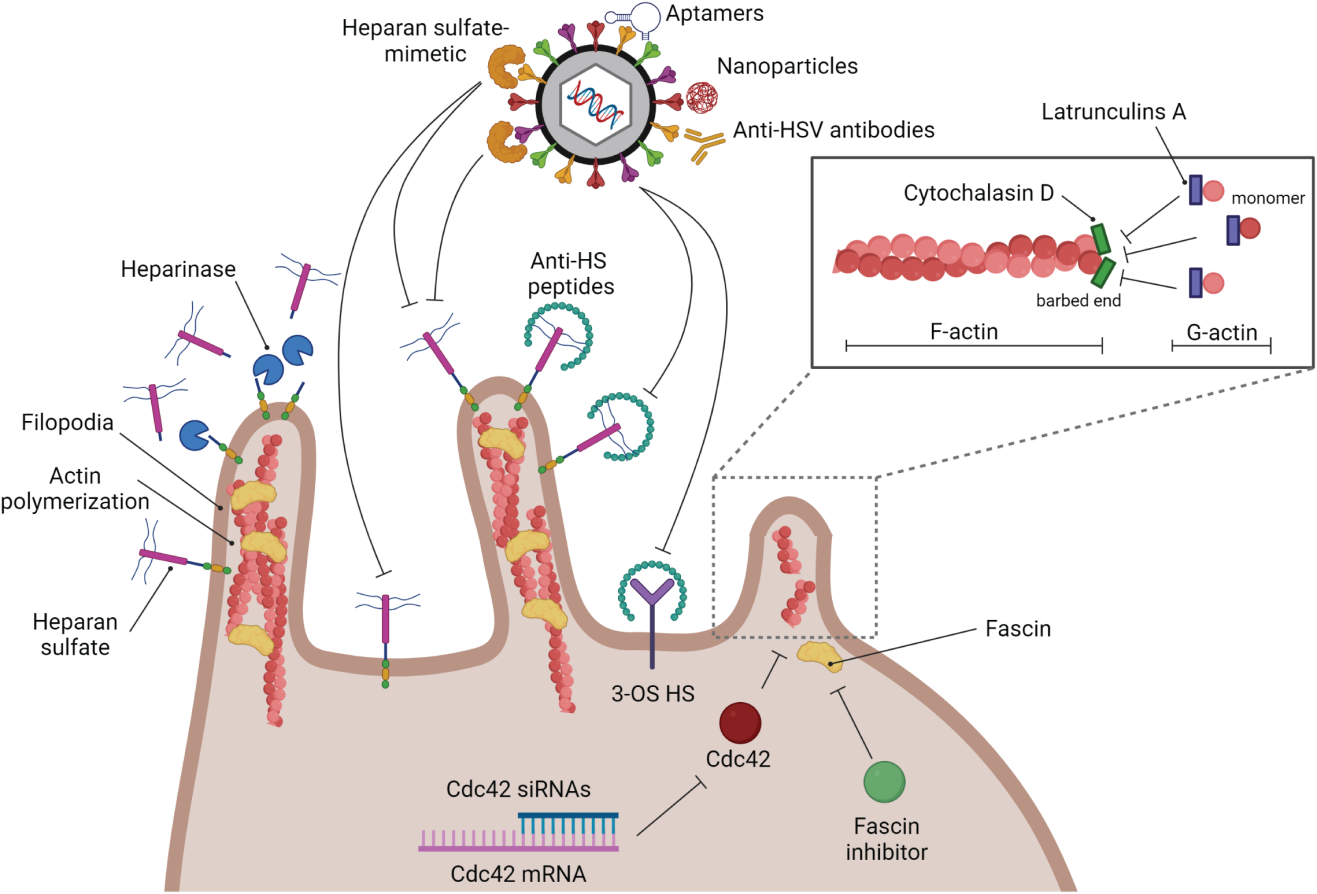
Strategies of anti-HSV drugs to block HSV at the entry step. Several kinds of substances have been developed to block HSV infection at the entry step. Blocking glycoprotein receptors; heparinase, and anti-HS peptides. Blocking viral glycoproteins directly; heparan sulfate-mimetic, aptamers, nanoparticles, and anti-HSV antibodies. Filopodia inhibition by actin polymerization disruption; cytochalasin D, and latrunculin A.

To this date, most of the anti-viral targets for HSV treatment are at the viral replication step. Blocking HSV at the entry step may be beneficial in some cases, especially in HSV drug-resistant infections. The strategies to block HSV entry are shown in **Figure 4**. To reduce HSV-1 viremia, it might be interesting to block HSV attachment on filopodia of T lymphocytes. Reducing viral entry by blocking this attachment step has previously been demonstrated in epithelial cells. Heparinase treatment provides a negative effect on HSV-1 entry since HSPG, which is widely expressed on filopodia, is removed after treatment (Immergluck et al., 1998). Tiwari et al. (2011) demonstrated that HSV-1 infection is blocked by anti-HS (heparan sulfate) peptides (Tiwari et al., 2011). Moreover, a similar result has been demonstrated by using the low-molecular-weight heparan sulfate-mimetic (Nyberg et al., 2004), pentosan polysulfate (Herold et al., 1997), dextran sulfate (Dyer et al., 1997), and sulfated maltoheptaose (Herold et al., 1997).

As mentioned previously, filopodia formation is a key structure that enhances HSV infectivity in many kinds of cells. Hence, destruction of this formation should suppress HSV entry. There are many types of small molecules that can interact with actin molecules to promote actin polymerization and depolymerization, which may affect the formation of filopodia. Phalloidins, a toxin of the death cap mushroom (*Amanita phalloides*), can support F-actin polymerization by inhibiting actin filament disassembly. Jasplakinolides, a cytotoxin that is isolated from the marine sponge *Jaspis splendens*, enhances actin filament assembly by stabilizing G-actin monomers (Watts et al., 2011). On the other hand, Latrunculin A and cytochalasin D, are actin-depolymerizing agents. Latrunculin A, a toxin produced by sponges (genus *Latrunculia* and *Negombata*), binds to G-actin monomers, inhibiting their polymerization activity and promoting F-actin disassembly (Morton et al., 2000; Yarmola et al., 2000). Cytochalasin (cyto) D, a mycotoxin produced by *Helminthosporium* and other molds, binds to the barbed (+) end of F-actin to prevent G-actin assembly and promote F-actin disassembly at that site. Many previous studies used cyto D to block filopodia formation and reduce the infectivity of some viruses. The amount of HSV-1 entry in CHO cells is decreased when the cells are treated with cyto D (Burnham et al., 2016). Cyto D treatment leading to inhibition of filopodia formation and viral surfing plays a critical role not only in epithelial cells but also in neurons and T lymphocytes (Dixit et al., 2008; Sasivimolrattana et al., 2021). However, blocking HSV entry by suppressing filopodia formation and interfering with the competitive binding of the gB receptor (HSPG) might not be enough to restrict complete HSV entry since there are other gB and gD receptors that normally express on the cell surface. Hence, this strategy should be co-treated with another kind of anti-HSV entry drugs or compounds. Note that actin is a key cytoskeletal component. Disruption of actin polymerization might also damage the cell. As a result, while these inhibitors are helpful for *in vitro* research, it doesn’t appear that they could be used in clinical settings. Hence, it is interesting to use another inhibitor of filopodia formation. A study demonstrated that Cdc42 knockdown by using siRNA targeted to Cdc42 suppressed the number of filopodia and the HSV entry rate in HeLa cells (Oh et al., 2010). Han S. and co-workers developed the fascin inhibitors, the main actin-bundling protein in filopodia, using fascin-specific small molecules to suppress the formation of filopodia, which blocked tumor cell migration and metastasis (Han et al., 2016). This molecule inhibits the interaction between fascin and actin (Han et al., 2016). However, there is no data about the use of fascin inhibitors to reduce HSV infection. On the other hand, to avoid the side effects of blocking host molecules and some signaling pathways, inhibiting HSV entry by using HSV virion as a target has been developed in many studies. The various anti-HSV entry approaches, including anti-HSV peptides, anti-HSV antibodies, aptamers, and nanoparticles, have been reviewed by Madavaraju K., et al. (Madavaraju et al., 2020).

## 4 Conclusions

Overall, the research findings discussed in this review demonstrated that HSV-1 can enter several cell types in a similar manner, i.e., epithelial, neuronal, and T lymphocytes. Actin polymerization and filopodia formation play critical roles in the enhancement of HSV-1 entry. Although many anti-viral drugs for HSV treatment, e.g., acyclovir and penciclovir, are very effective in inhibiting HSV replication, some HSV anti-viral drug resistance is still present. According to this review, it is interesting to use the entry step as an alternative target for anti-HSV drugs. As mentioned, there are many strategies to block HSV entry, for example, heparinase treatment, anti-HS peptides, and low-molecular-weight heparan sulfate-mimetic. Taken together, further studies should be conducted to find a solid alternative platform to block HSV infection. Moreover, uncovering the cellular and molecular mechanisms underlying HSV infection and filopodia formation is therefore critical to identifying novel targets for therapy and gaining a better understanding of the viral pathogenesis.

## Author contributions

TS: Conceptualization, Funding acquisition, Project administration, Software, Writing – original draft. PB: Conceptualization, Project administration, Supervision, Writing – review & editing.
